# Comparing Single and Dual Console Systems in the Robotic Surgical Training of Graduating OB/GYN Residents in the United States

**DOI:** 10.1155/2016/5190152

**Published:** 2016-02-03

**Authors:** Emad Mikhail, Jason L. Salemi, Stuart Hart, Anthony N. Imudia

**Affiliations:** ^1^Department of Obstetrics and Gynecology, University of South Florida, Morsani College of Medicine, Tampa, FL 33606, USA; ^2^Department of Family and Community Medicine, Baylor College of Medicine, Houston, TX 77098, USA

## Abstract

*Objective*. To assess the impact of a single versus dual console robotic system on the perceptions of program directors (PD) and residents (RES) towards robotic surgical training among graduating obstetrics and gynecology residents.* Design*. An anonymous survey was developed using* Qualtrics*, a web-based survey development and administration system, and sent to obstetrics and gynecology program directors and graduating residents.* Participants*. 39 program directors and 32 graduating residents (PGY4).* Results*. According to residents perception, dual console is utilized in about 70% of the respondents' programs. Dual console system programs were more likely to provide a robotics training certificate compared to single console programs (43.5% versus 0%, *p* = 0.03). A greater proportion of residents graduating from a dual console program perform more than 20 robotic-assisted total laparoscopic hysterectomies, 30% versus 0% (*p* = 0.15).* Conclusions*. Utilization of dual console system increased the likelihood of obtaining robotic training certification without significantly increasing the case volume of robotic-assisted total laparoscopic hysterectomy.

## 1. Introduction

Despite recent increases in the use of robotic surgical systems in gynecologic procedures, a trend that is expected to continue [1], incorporation of robotics into the training environment has been difficult because of the one-surgeon and one-surgery limitations of the robotic system [2]. As the transition is made into this “robotic era,” in which only one surgeon at a time can perform any given operation, it is essential to provide appropriate training and ensure the competence of trainees before they literally take over the controls [3]. Some robotic surgery systems have a single surgeon console while other systems have an additional mentoring console (dual console). The dual console system has the potential to improve resident participation and training in robotic-assisted surgical cases, which is likely to translate into improved patient safety during robotic surgery [4].

Although the learning curve for robotic surgery may be less steep than for conventional laparoscopic surgery [5], the depth of residents' participation in the operating room has a strong impact on their surgical aptitude. Robotic gynecologic surgery has been associated with increased postoperative complications when compared to laparoscopic gynecologic surgery [6], which may, in part, be due to deficiencies in surgical training. In order to address the increased morbidity associated with robotic gynecologic surgeries, many residency programs across the nation have incorporated specific robotic training curriculum and some hospitals have purchased newer robotic systems with dual console. Collectively, these measures are aimed at increasing trainees' exposure and skills in robotic gynecologic surgeries. No study to date has evaluated the impact of the introduction of the dual console robotic system in residency robotic surgical training. Therefore, the objective of this study is to assess the perceptions of program directors (PD) and graduating obstetrics and gynecology residents (RES) towards robotic surgical training and to determine whether the existence of a single versus dual console robotic system as part of the residency program impacts those perceptions.

## 2. Materials and Methods

A survey was developed using* Qualtrics*, a web-based survey development and administration system. After obtaining exempt status from University of South Florida Institutional Review Board, the survey was pilot tested to ensure that it was easy to understand and that questions were relevant and to gauge the time required to complete the survey. The survey was sent by email to all obstetrics and gynecology residency program directors (*n* = 243). The list of emails was obtained from Association of Professors of Gynecology and Obstetrics (APGO) website. The email survey was also sent to the listserv of the obstetrics and gynecology residency program coordinators, and a request was made to forward the survey to all graduating (PGY4) obstetrics and gynecology residents in their program (*n* = 1255). After sending the initial email, two additional reminders were sent to improve the likelihood of response. The first section of the survey focused on demographics, the location and type of residency, and the number of residents in the program per year. The second section focused on aspects of individual training and surgical volume and personal perspective on robotic utilization in gynecologic surgery.

Survey responses were analyzed using SAS, version 9.4 (SAS Institute, Inc., Cary, NC). Frequencies and percentages of responses were generated overall, by type of respondent (PD, RES) and by type of robotic system (single, dual). Since the main study question sought to determine whether responses varied according to the presence of a single or dual robotic system in the program, Fisher's exact tests (characteristics with 2 groups) or Freeman-Halton tests (characteristics with >2 groups) were used to assess crude associations between each individual/program characteristic and type of robotic system. A *p* value of <0.05 is considered statistically significant.

## 3. Results

A total of 84 participants accessed the survey; 13 were missing responses for more than 50% of questions, including the type of robotic system, and were excluded from the analysis. There were 39 PD and 32 RES completed surveys. The response rates were 16% and 2.5% for the PD and RES, respectively. Nearly 94% of RES respondents were female, compared to only 66.7% of PD. The majority of RES were in a university program (71.9%) compared to 53.8% of PD. Nearly half of PD felt comfortable that residents from their program could perform robotic-assisted total laparoscopic hysterectomy (RA-TLH) upon graduation without the need for proctoring, despite their reporting that only 28.2% of their residents performed more than 20 RA-TLH before graduating. Similarly, 53.1% of RES respondents felt comfortable that residents from their program could perform RA-TLH upon graduation without proctoring even though only about 1 in 5 performed more than 20 RA-TLH before graduating. RES and PD shared the same belief that utilization of robotic surgery will increase in gynecologic oncology but probably will either decrease or stay the same in other gynecologic surgery subspecialties, Table 1.

Thirty PD (76.9%) and 23 RES (71.9%) report having a dual console in their hospital. Among PD respondents—although not statistically significant—university programs are more likely to utilize a dual console system than community or university-affiliated community programs (*p* = 0.66). Regardless of the subspecialty, programs with dual console tended to offer more subspecialty fellowships. The programs with dual console system tended to provide a robotic training certificate more frequently, 50% versus 22% (*p* = 0.15), and a bigger proportion of their graduating residents performed more than 20 RA-TLH, 33% versus 11% (*p* = 0.39). The presence of dual console did not significantly change PD perspectives on future utilization of robotics in different subspecialty of gynecologic surgery, Table 2.

According to RES responses, more university programs tend to utilize a dual console system (*p* = 0.20). The presence of dual console did not increase the proportion of the programs that offer subspecialty fellowship training. Surprisingly, 44% of single console programs offer minimally invasive gynecologic surgery fellowship compared to only 34.8 of dual console programs (*p* = 0.70). Dual console system programs were more likely to provide a robotics training certificate compared to single console programs (43.5% versus 0%, *p* = 0.03%). A greater proportion of residents graduating from a dual console program perform more than 20 RA-TLH, 30% versus 0% (*p* = 0.15). The presence of a dual console did not significantly change the RES perceptions of the future utilization of robotics in various gynecologic surgery subspecialties, Table 3.

**Figure 1:**
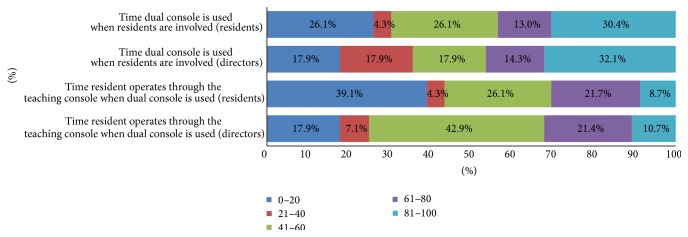
Involvement of residents during robotic operations using the dual console, based on perceptions of graduating obstetrics and gynecology residents and program directors.

The dual console is generally underutilized; only 30% of PD and RES report that the dual console is utilized more than 80% of the time in cases with resident involvement, while more than 50% of RES and PD report that the dual console is utilized less than 60% of the time. Moreover, only 10.7% of PD report that the residents operate through the teaching console more than 80% of the time compared to only 8.7% of RES, while about 70% of RES and PD report that the residents operate through the teaching console less than 60% of the time, Figure 1.

## 4. Discussion

This pilot survey study showed that robotic surgical training was comparable with and without the utilization of the dual console. As far as the graduating residents are concerned, the presence of a dual console system was influential in obtaining a postresidency training certificate. We acknowledge that our survey response rate is low regarding both PD and RES, but since this is the first study to address the perception for differences in robotic surgical training between single and dual console systems, we believe that our findings are thought provoking and stimulate the need for future studies. The da Vinci Surgical System® (Intuitive Surgical Corporations, Sunnyvale, California, USA) was first approved by the Food and Drug Administration for gynecologic surgeries in April 2005, and Intuitive Surgical introduced the da Vinci Si dual console interface in April 2009, allowing 2 surgeons to sit at 2 different surgical consoles and control the same robot simultaneously [7].

This study is the first to compare residents' and program directors' perception of robotic surgical training with and without the dual console system. It is known that a major hurdle to success in robotic surgery is the associated learning curve, which applies to both the surgeon and the surgical team. Utilization of the dual console system might give a second surgeon the opportunity to gain robotic experience, which in turn may result in earlier proficiency [2]. A study by Crusco et al. concluded that there were no significant differences observed in performance time when teaching knot-tying techniques to novice medical students using the da Vinci dual console compared with the single console [7]. Although the aforementioned study focused on training medical students, its results were similar to the results of this survey. No significant difference was found regarding number of RA-TLH performed or the level of comfort performing RA-TLH without proctoring.

An advantage of dual console is that the trainee's perception of being removed from the procedure almost disappears [8]. The use of the dual console enables integrated teaching, surgical cooperation with proctoring, and supervision, without compromising operative times or patient outcomes [2]. The give or take function refers to each instrument in use, the control of which can be given singularly to the learning surgeon. The swap all function allows the master surgeon to gain full control of all the instruments at 1 time. This console also allows the learning surgeon to operate the robot in a “simplified” fashion, with 2 operating arms, while the proctor surgeon controls the third arm for retraction, exposure, or even pointing [8]. The virtual pointer is another useful tool of the dual console that facilitates surgical training. It enables the operator or proctor to point and refer to specific anatomic features on the live video image intraoperatively. Finally, there is also the emergency stop button, which can be pressed at any time, should the situation require a sudden cessation of activity [8]. The disadvantages of dual console include the increased cost and requirement of extra manpower, because another assistant surgeon is needed at the patient table to maneuver the instruments via the assistant ports, exchange the robotic instruments, and troubleshoot for glitches related to the robotic platform [8].

In this study, the dual console system is available in about 70% of the OBGYN residency programs that participated in the survey. A study published by Gobern et al. showed that robotic platforms were available at 82% of institutions associated with a residency program. Robotic-assisted gynecologic procedures were performed at 78% of these institutions. Resident training in robotic surgery, however, was part of the training curriculum at only 58% of those residency programs [1]. Despite that, the utilization of the dual console is variable, with 50% of our study participants reporting that the dual console is utilized less than 60% of the time.

The effect of surgical volume on perioperative outcomes and resource utilization is evident. In a study by Wallenstein et al., women treated by high-volume surgeons and at high-volume hospitals have lower morbidity; also surgical volume has a direct effect on lowering resource utilization [9]. In a study by Pitter et al., a significant improvement in operating time was noted after 20 hysterectomy and myomectomy cases were performed [10]. Despite that, defining a cutoff threshold is still difficult [11]. Surgeon volume is increasingly being used as a component in assessment of individual and hospital surgical quality [11]. Meanwhile, organizations including the American Association of Gynecologic Laparoscopists (AAGL) propose that a minimum of 20 cases per year is needed to maintain robotic privileges [12]. In this study, a greater proportion of residents graduating from a dual console program performed more than 20 RA-TLH compared to single console program, but the difference was not statistically significant [30% versus 0% (*p* = 0.15)].

It seems from this study that the availability of a dual console system does not add a significant benefit in the robotic training of the residents except that programs with a dual console tend to award more robotic training certificates to their graduating residents. However, there are some limitations such as recall, response, and research biases, which are inherent to the design of survey studies. Our response rates were 16% and 2.5% for the PD and RES, respectively, despite sending two reminder emails to encourage better response. We acknowledge that this is a major limitation of our study. Additionally, we acknowledge that this study might be underpowered to find statistically significant differences between the single and the dual console groups. Therefore, future studies with more participants and analysis of perioperative outcomes as primary endpoint between the two groups will be needed to definitely determine the utility of dual console robotic system in residency training.

## Figures and Tables

**Table 1:** Characteristics of residency programs, based on perceptions of residency program directors and of graduating obstetrics and gynecology residents.

Characteristic	Survey participant	
	Program directors (*n* = 39)	Residents (*n* = 32)
Female	26 (66.7)	30 (93.8)
Age		
<35 years	0 (0.0)	31 (96.9)
35–44 years	14 (35.9)	1 (3.1)
45–54 years	15 (38.5)	0 (0.0)
55–64 years	9 (23.1)	0 (0.0)
Residency program type		
University	21 (53.8)	23 (71.9)
University-affiliated community	11 (28.2)	5 (15.6)
Community	6 (15.4)	4 (12.5)
Program has more than 5 residents per year^a^	19 (48.7)	23 (71.9)
Fellowships sponsored	20 (51.3)	22 (68.8)
Gynecologic oncology	9 (23.1)	11 (34.4)
Female pelvic medicine and reconstructive surgery	11 (28.2)	11 (34.4)
Minimally invasive gynecologic surgery	6 (15.4)	12 (37.5)
Reproductive endocrinology and infertility	10 (25.6)	10 (31.3)
Maternal fetal medicine	16 (41.0)	20 (62.5)
Robotic training certificate available	17 (43.6)	10 (31.3)
Graduating resident perform >20 RA-TLH^b^	11 (28.2)	7 (21.9)
Feeling comfortable that residents from program can perform RA-TLH upon graduation without proctoring	19 (48.7)	17 (53.1)
Future utilization of robotic surgery will increase in^c^		
Benign gynecologic surgery	12 (30.8)	9 (28.1)
Reconstructive pelvic surgery	17 (43.6)	17 (53.1)
Gynecologic oncology	26 (66.7)	27 (84.4)
Reproductive surgery	12 (30.8)	13 (40.6)

RA-TLH = robotic-assisted total laparoscopic hysterectomy.

^a^Comparison group consists of programs with 5 or fewer residents per year.

^b^Comparison group consists of programs in which residents perform 20 or fewer RA-TLH.

^c^Comparison group for each category is a response that utilization of robotic surgery will stay the same or decreases.

**Table 2:** Characteristics of residency programs with single versus dual console robotic systems, based on perceptions of residency program directors.

Characteristic	Does the residency programs have a dual console robotic system?		*p* value^a^
	Yes (*n* = 30)	No (*n* = 9)	
Female	18 (60.0)	8 (88.9)	0.22
Age			0.26
35–44 years	11 (36.7)	3 (33.3)	
45–54 years	13 (43.3)	2 (22.2)	
55–64 years	5 (16.7)	4 (44.4)	
Residency program type			0.66
University	17 (56.7)	4 (44.4)	
University-affiliated community	8 (26.7)	3 (33.3)	
Community	4 (13.3)	2 (22.2)	
Program has more than 5 residents per year^b^	16 (53.3)	3 (33.3)	0.45
Fellowships sponsored			
Gynecologic oncology	8 (26.7)	1 (11.1)	0.65
Female pelvic medicine and reconstructive surgery	9 (30.0)	2 (22.2)	0.99
Minimally invasive gynecologic surgery	5 (16.7)	1 (11.1)	0.99
Reproductive endocrinology and infertility	9 (30.0)	1 (11.1)	0.40
Maternal fetal medicine	14 (46.7)	2 (22.2)	0.26
Robotic training certificate available	15 (50.0)	2 (22.2)	0.15
Graduating resident perform >20 RA-TLH^c^	10 (33.3)	1 (11.1)	0.39
Feeling comfortable that residents from program can perform RA-TLH upon graduation without proctoring	15 (50.0)	4 (44.4)	0.99
Future utilization of robotic surgery will increase in^d^			
Benign gynecologic surgery	10 (33.3)	2 (22.2)	0.99
Reconstructive pelvic surgery	15 (50.0)	2 (22.2)	0.42
Gynecologic oncology	21 (70.0)	5 (55.6)	0.99
Reproductive surgery	11 (36.7)	1 (11.1)	0.39

RA-TLH = robotic-assisted total laparoscopic hysterectomy.

^a^ *p* value from Fisher's exact test (characteristics with 2 groups) or Freeman-Halton test (characteristics with >2 groups).

^b^Comparison group consists of programs with 5 or fewer residents per year.

^c^Comparison group consists of programs in which residents perform 20 or fewer RA-TLH.

^d^Comparison group for each category is a response that utilization of robotic surgery will stay the same or decreases.

**Table 3:** Characteristics of residency programs with single versus dual console robotic systems, based on perceptions of graduating obstetrics and gynecology residents.

Characteristic	Does the residency programs have a dual console robotic system?		*p* value^a^
	Yes (*n* = 23)	No (*n* = 9)	
Female	21 (91.3)	9 (100)	0.99
Age < 35 years	22 (95.7)	9 (100)	0.99
Residency program type			0.20
University	18 (78.3)	5 (55.6)	
University-affiliated community	2 (8.7)	3 (33.3)	
Community	3 (13.0)	1 (11.1)	
Program has more than 5 residents per year^b^	16 (69.6)	7 (77.8)	0.99
Fellowships sponsored			
Gynecologic oncology	8 (34.8)	3 (33.3)	0.99
Female pelvic medicine and reconstructive surgery	8 (34.8)	3 (33.3)	0.99
Minimally invasive gynecologic surgery	8 (34.8)	4 (44.4)	0.70
Reproductive endocrinology and infertility	9 (39.1)	1 (11.1)	0.21
Maternal fetal medicine	15 (65.2)	5 (55.6)	0.70
Robotic training certificate available	10 (43.5)	0 (0)	**0.03**
Graduating resident perform >20 RA-TLH^c^	7 (30.4)	0 (0)	0.15
Feeling comfortable that residents from program can perform RA-TLH upon graduation without proctoring	14 (60.9)	3 (33.3)	0.41
Future utilization of robotic surgery will increase in^d^			
Benign gynecologic surgery	8 (34.8)	1 (11.1)	0.38
Reconstructive pelvic surgery	11 (47.8)	6 (66.7)	0.19
Gynecologic oncology	19 (82.6)	8 (88.9)	0.55
Reproductive surgery	9 (39.1)	4 (44.4)	0.64

RA-TLH = robotic-assisted total laparoscopic hysterectomy.

^a^ *p* value from Fisher's exact test (characteristics with 2 groups) or Freeman-Halton test (characteristics with >2 groups).

^b^Comparison group consists of programs with 5 or fewer residents per year.

^c^Comparison group consists of programs in which residents perform 20 or fewer RA-TLH.

^d^Comparison group for each category is a response that utilization of robotic surgery will stay the same or decreases.
